# Mapping developmental QTL for plant height in soybean [*Glycine max* (L.) Merr.] using a four-way recombinant inbred line population

**DOI:** 10.1371/journal.pone.0224897

**Published:** 2019-11-20

**Authors:** Hong Xue, Xiaocui Tian, Kaixin Zhang, Wenbin Li, Zhongying Qi, Yanlong Fang, Xiyu Li, Yue Wang, Jie Song, Wen-Xia Li, Hailong Ning

**Affiliations:** 1 Key Laboratory of Soybean Biology, Ministry of Education, Harbin, China; 2 Key Laboratory of Soybean Biology and Breeding / Genetics, Ministry of Agriculture, Harbin, China; 3 College of Crop Science, Northeast Agricultural University, Harbin, Heilongjiang province, China; 4 Keshan Branch of Heilongjiang Academy of Agricultural Sciences, Keshan,Heilongjiang, China; North Dakota State University, UNITED STATES

## Abstract

Plant height (PH) is an important trait in soybean, as taller plants may have higher yields but may also be at risk for lodging. Many genes act jointly to influence PH throughout development. To map the quantitative trait loci (QTL) controlling PH, we used the unconditional variable method (UVM) and conditional variable method (CVM) to analyze PH data for a four-way recombinant inbred line (FW-RIL) population derived from the cross of (Kenfeng14 × Kenfeng15) × (Heinong48 × Kenfeng19). We identified 7, 8, 16, 19, 15, 27, 17, 27, 22, and 24 QTL associated with PH at 10 developmental stages, respectively. These QTL mapped to 95 genomic regions. Among these QTL, 9 were detected using UVM and CVM, and 89 and 66 were only detected by UVM or CVM, respectively. In total, 36 QTL controlling PH were detected at multiple developmental stages and these made unequal contributions to genetic variation throughout development. Among 19 novel regions discovered in our study, 7 could explain over 10% of the phenotypic variation and contained only one single QTL. The unconditional and conditional QTL detected here could be used in molecular design breeding across the whole developmental procedure.

## Introduction

Soybean [*Glycine max* (L.) Merri.] is an important source of protein and edible oil [1]. Among all breeding traits, plant height (PH) is of importance for it enhances the seed yield by increasing pod-bearing and decreasing the degree of lodging. Genetically, PH is a quantitative trait that is controlled by multiple genes [2]. In the past decades, many studies have focused on mapping the quantitative trait loci (QTL) associated with PH in soybean [3–18]. At present, 255 QTL underlying PH have been integrated on the public genetic linkage map (http://www.soybase.org/). The QTL in soybean were identified mainly based on phenotypic values at the mature stage [2–7], whereas only 22 QTL were mapped based on the development of PH throughout the growing season [8]. PH continues to develop from emergence to the end of flowering and involves multiple genes. Therefore, in to gain a comprehensive understanding of the genetic basis of a trait such as PH, it is necessary to understand the dynamics of gene expression for the trait at different developmental stages [19]. Indeed, QTL mapping based on the final stage of development may not encompass the numerous QTL controlling PH during the earlier developmental stages and may not show genetic effects within a specific stage of plant development [20].

The detection of dynamic QTL throughout development could lead to a better understanding of the developmental effects of quantitative traits, and such QTL would be useful for marker-assisted selection programs [21]. The development of morphological traits occurs through the action of individual genes and the interaction of multiple genes [22]. The gene expression differs during various growth stages and is modified by gene interactions as well as the interaction between genes and the environment [23]. To demonstrate the complex process of trait development, a genetic model was established to evaluate the net genetic effects of quantitative traits during specific developmental stages [24]. Recently, dynamic QTL mapping has been successfully used to dissect the influence of quantitative traits in cotton (*Gossypium* sp.) [25], rice (*Oryza sativa*) [26–31], maize (*Zea mays*) [32], and wheat (*Triticum* sp.) [33–35] throughout development. In soybean, the dynamic QTL underlying PH [8], seed composition [36], reproductive growth stages [37], seed weight [38–39], seed protein content [21], linolenic acid content [40], and seed mass filling rate [19] have been investigated.

All of the above findings were obtained based on lines derived from an initial cross between two inbred lines. In a bi-parental mapping population, a single locus has only two alleles, and only QTL that are segregating between the two parental lines can be detected, and therefore, the statistical inference space is more narrow [41]. Thus, the QTL detection efficiency of bi-parental crosses is significantly less than in multi-parental populations because of the lack of genetic diversity and other limitations [42–46]. In contrast, a four-way cross (FWC) involves four inbred lines, which can be inferred on a wider statistical inference space than a simple two-line cross, and therefore improves the efficiency of QTL detection [44–48]. In crop breeding, a double cross, three-way cross, and other hybridization methods involving multiple parents are often used for breeding varieties or parental lines. A FWC involves four inbred lines (L1, L2, L3, and L4) and can be expressed as (L1 × L2) × (L3 × L4). Xu (1996) reported a QTL mapping method using a FWC, which improves the QTL detection efficiency [43]. FWC populations have been studied in cotton [48], *Arabidopsis thaliana* [49], mice [50], and pigs [51].

In our previous work, we produced a four-way recombinant inbred line (FW-RIL) population and mapped QTL for quality and pod number traits in soybean [52–53]. In the present study, we investigated PH in soybean throughout its development to detect conditional and unconditional QTL based on four environments (four different sowing dates) using our FW-RIL population comprising 160 F_2:7_ and F_2:8_ individuals derived from the cross (Kenfeng14 × Kenfeng15) × (Heinong48 × Kenfeng19). The objective of our study was to identify QTL for PH throughout development in soybean, and to propose a molecular design for improving PH.

## Materials and methods

### Genetic population

Four soybean varieties namely Kenfeng14, Kenfeng15, Heinong48 and Kenfeng19 were used to produce a FWC population. The germplasm of these four parents showed extensive genetic variation and significant phenotypic variation for PH (S1 Table). Two single crosses, Kenfeng14 × Kenfeng15 and Heinong48 × Kenfeng19 were conducted in the spring of 2008 in Harbin, and 21 and 32 F_1_ hybrid seeds were harvested from the two crosses, respectively. All F_1_ seeds of the two crosses were sown and crossed to produce a double cross hybrid (Kenfeng14 × Kenfeng15) × (Heinong48 × Kenfeng19) in 2009, and 175 F_1_ seeds were obtained. All the seeds were sown and selfed for five generations continuously from 2010 to 2013 near Harbin (126.63°E, 45.75°N), Heilngjiang Province and near Yacheng (109.00°E, 17.5°N), Hainan Province, China, and each individual was picked by single-seed descent at maturity stage. Finally, 160 FW-RILs were obtained and used for construction of a genetic linkage map and QTL identification.

### Sowing date, experimental design, and field experiment

The RILs and the parental lines were grown in a sequential design with two replications at Harbin in Heilongjiang Province in China. The seeds were sown on May 3 (E1) and 25 (E2) in 2014 and May 9 (E3) and 25 (E4) in 2015. Seeds were sown along a single line in each row of three-row plots of 5 m in length, with 0.67 m between rows, and 0.07 m spacing between plants within the rows. The field management practices were similar to those of local soybean production. The soil type was fine-mesic chernozem soil, which contained 199 ± 23.0 mg·kg^-1^ of available potassium, 52 ± 1.9 mg·kg^-1^ of available phosphorus, 116 ± 6.8 mg·kg^-1^ of alkali-hydrolyzable nitrogen, and a pH of 6.8.

### Collection of phenotypic data

For each sowing period, five consecutive plants from the middle row of the plot, which had the same genotypes, were sampled for the experiment. PH (length from the cotyledonary node to the top of the plants) was measured every seven days until PH reached a maximum, from 7 days after emergence (DAE) to 70 DAE for the first sowing period (E1) and for the second sowing period (E2) in 2014, and 14 DAE to 70 DAE for the first sowing period (E3) and 14 DAE to 63 DAE for the second sowing period (E4) in 2015. Emergence was determined, as the day in which over 50% of the plants had emerged in a plot.

### Data analysis

#### Conditional variables analysis

The unconditional variable method (UVM) and conditional variable method (CVM) were used to analyze the cumulative effect from the initial to *t*^*th*^ stage and the net effect from *t*−1^*th*^ stage to *t*^*th*^ stage. The unconditional variable values were the phenotype, and the conditional values referred to the net effect from the (*t*−1)^*th*^ stage to the *t*^*th*^ stage. Specifically, the unconditional values analysis was conducted depending on the average in each plot. The conditional values were estimated via the method proposed by Zhu [24] with the following formula:

$$y_{t|t-1}=y_{t}-C_{t,t-1}\cdot (y_{t-1}-{\bar{y}}_{t-1})/V_{t-1}$$

Where, *y*_*t*|*t*−1_ is the conditional value of the phenotype of the *t*^*th*^ stage on *t*−1^*th*^. *y*_*t*_ and *y*_*t*−1_ are the phenotype of the individual at *t* and *t*−1 stages, respectively, while ${\bar{y}}_{t-1}$ is the mean values of the phenotype at the *t*−1 stage. *C*_*t*,*t*−1_ is the covariance between the phenotype at *t* and *t*−1 stages. *V*_*t*−1_ is the variance of the phenotype at the *t*−1 stage. The calculation of conditional values was conducted via this formula in Microsoft Excel 2010.

#### Variation analysis and heritability of phenotypic traits

Based on unconditional and conditional values of the three replicates for each line in each sowing date, summarization, analysis of variance (ANOVA), correlation analysis, and heritability for each environment were conducted.

ANOVA for each environment was carried out based on following statistical model:

$$x_{ij}=\mu +G_{i}+R_{j}+\varepsilon_{ij}$$

Where, *x*_*ij*_ is the observed value of the *i*th genotype in the *j*th block in some environment, *μ* is the grand mean, *G*_*i*_ is the effect of the *i*th genotype, *R*_*j*_ is the effect of the *j*th block, and *ε*_*ij*_ is the residual error of the *i*th genotype in the *j*th block, *ε*_*ij*_ ~ *N*(0, $\sigma_{\varepsilon }^{2}$).

Heritability for each environment was estimated through the following formula:

$$h^{2}=\frac{\sigma_{G}^{2}}{\sigma_{G}^{2}+\sigma_{\varepsilon }^{2}}$$

Where, *h*^2^ indicates the broad sense heritability, $\sigma_{G}^{2}$ indicates the genetic variance, $\sigma_{\varepsilon }^{2}$ indicates the residual variance. All calculations were implemented using SAS 9.2 software (SAS Institute Inc., USA).

#### QTL mapping

Based on the simple sequence repeat (SSR) linkage map constructed in our previous research [52–53], the unconditional and conditional data of the four different sowing dates were used to conduct QTL analysis by the inclusive composite interval mapping (ICIM) method [54]. Construction of linkage groups and mapping QTL was implemented through GAPL V1.0 software (www.isbreeding.net). The probability for the selection of markers to correct the background error was set at 0.0001; the step of one-dimensional scanning for QTL was set at 1 cM. The log of the odds (LOD) was produced by permutation methods (1000 iterations and the probability of first type error of 0.05), and the least value was set at 2.5 to declare the existence of QTL.

The QTL were named following the method proposed by McCouch [55].

## Results

### Phenotypic variation

**Fig 1:**
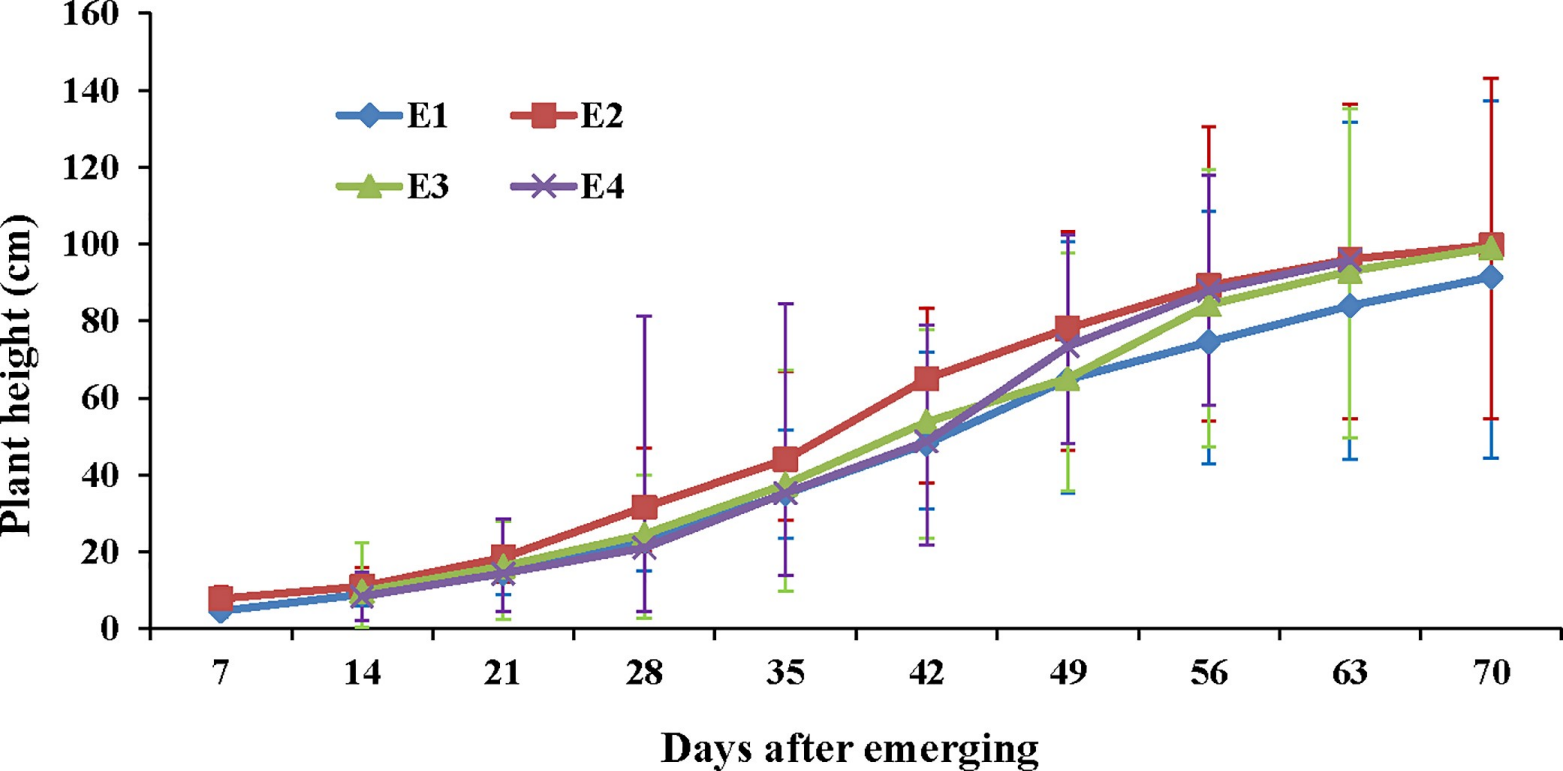
Development of plant height in the four environments. E1 represents the May 3, 2014 sowing date; E2 represents the May 25, 2014 sowing date; E3 represents the May 9, 2015 sowing date; E4 represents the May 25, 2015 sowing date; The top, middle, and bottom bar with the same color show the maximum, mean, and minimum values for one stage; The color picture can be downloaded in dio 10.1371/journal.pone.0224897.

At each developmental stage and four different sowing dates over a period of two years, the frequency of PH of the FW-RILs followed a normal distribution. Moreover, the PH values of the four parents were included within the range of the FW-RILs, which showed transgressive segregation in PH (S1 Fig). The average and range of PH gradually increased in the FW-RILs throughout the growth period (Fig 1), and PH exhibited an approximate “S” curve and stopped at 70 DAE for all 160 lines in each environment. There were significant variations among environment, genotype, and genotype×environment interaction effects at each stage (Table 1), which showed the changing of the genetic basis of PH in the whole development.

**Table 1:** Analysis of variance and heritability of each stage based on unconditional and conditional variables across the four environments.

Environment ^a^	Parameters ^b^	Traits ^c^																			
		DAE07		DAE14		DAE21		DAE28		DAE35		DAE42		DAE49		DAE56		DAE63		DAE70	
**E1**	V_G_	0.49	** ^d^	1.85	**	5.35	**	15.05	**	34.33	**	68.54	**	97.57	**	156.23	**	225.70	**	324.65	**
	h^2^	0.53		0.88		0.89		0.85		0.90		0.88		0.90		0.95		0.96		0.97	
**E2**	V_G_	1.46	**	3.47	**	8.95	**	26.61	**	48.23	**	65.33	**	86.09	**	151.56	**	195.04	**	260.44	**
	h^2^	0.77		0.93		0.91		0.92		0.92		0.86		0.91		0.94		0.95		0.97	
**E3**	V_G_			2.88	**	12.36	**	19.14	**	46.97	**	101.12	**	114.68	**	158.00	**	208.97	**	306.14	**
	h^2^			0.92		0.94		0.88		0.92		0.91		0.92		0.95		0.94		0.97	
**E4**	V_G_			3.89	**	6.64	**	14.61	**	81.55	**	68.95	**	130.76	**	126.04	**	159.84	**		
	h^2^			0.94		0.91		0.86		0.95		0.88		0.94		0.93		0.94			
**Com.**	V_E_	5.16	**	1.31	**	3.95	**	20.95	**	17.12	**	60.34	**	40.89	**	42.68	**	34.40	**	19.10	**
	V_G_	0.27	**	0.77	**	2.30	**	5.41	**	10.66	**	15.82	**	26.01	**	63.23	**	92.76	**	193.27	**
	V_GE_	0.73	**	2.25	**	6.03	**	13.44	**	42.11	**	60.21	**	81.25	**	84.71	**	104.61	**	103.81	**
	h^2^	0.32		0.55		0.58		0.58		0.48		0.47		0.53		0.73		0.76		0.84	
				DAE14|07		DAE21|14		DAE28|21		DAE35|28		DAE42|35		DAE49|42		DAE56|49		DAE63|56		DAE70|63	
**E1**	V_G_			1.49	**	1.80	**	6.34	**	17.45	**	18.01	**	39.37	**	30.33	**	44.07	**	42.53	**
	h^2^			0.84		0.74		0.76		0.84		0.70		0.87		0.83		0.89		0.93	
**E2**	V_G_			1.87	**	4.92	**	12.20	**	14.13	**	27.95	**	41.42	**	44.95	**	45.22	**	12.91	**
	h^2^			0.85		0.90		0.85		0.82		0.79		0.89		0.88		0.89		0.77	
**E3**	V_G_					8.08	**	6.54	**	12.53	**	60.18	**	37.21	**	95.71	**	48.35	**	44.67	**
	h^2^					0.93		0.75		0.81		0.89		0.86		0.95		0.89		0.93	
**E4**	V_G_					2.56	**	5.65	**	45.62	**	37.40	**	74.77	**	62.93	**	59.41	**		
	h^2^					0.83		0.70		0.94		0.85		0.93		0.91		0.93			
**Com.**	V_E_			2.15	**	3.95	**	21.44	**	17.63	**	63.14	**	44.17	**	43.56	**	32.16	**	18.97	**
	V_G_			0.03	**	0.28	**	0.35	**	2.14	**	0.00	**	8.98	**	16.41	**	10.53	**	8.86	**
	V_GE_			1.66	**	4.06	**	7.31	**	20.29	**	36.12	**	39.32	**	42.04	**	38.73	**	24.50	**
	h^2^			0.05		0.19		0.13		0.27		0.00		0.44		0.58		0.49		0.81	

^a^ E1 represents the May 3, 2014 sowing date; E2 represents the May 25, 2014 sowing date; E3 represents the May 9, 2015 sowing date; E4 represents the May 25, 2015 sowing date; Com. represents the combination of the four environments.

^b^ V_G_, V_E_, and V_GE_ represent the variance of genotypes, environments, and genotype×environment interaction effects, respectively; h^2^ represents heritability.

^c^ DAE represents plant height at days after emerging.

^d^ ** represents significant at < 0.0001 level.

**Fig 2:**
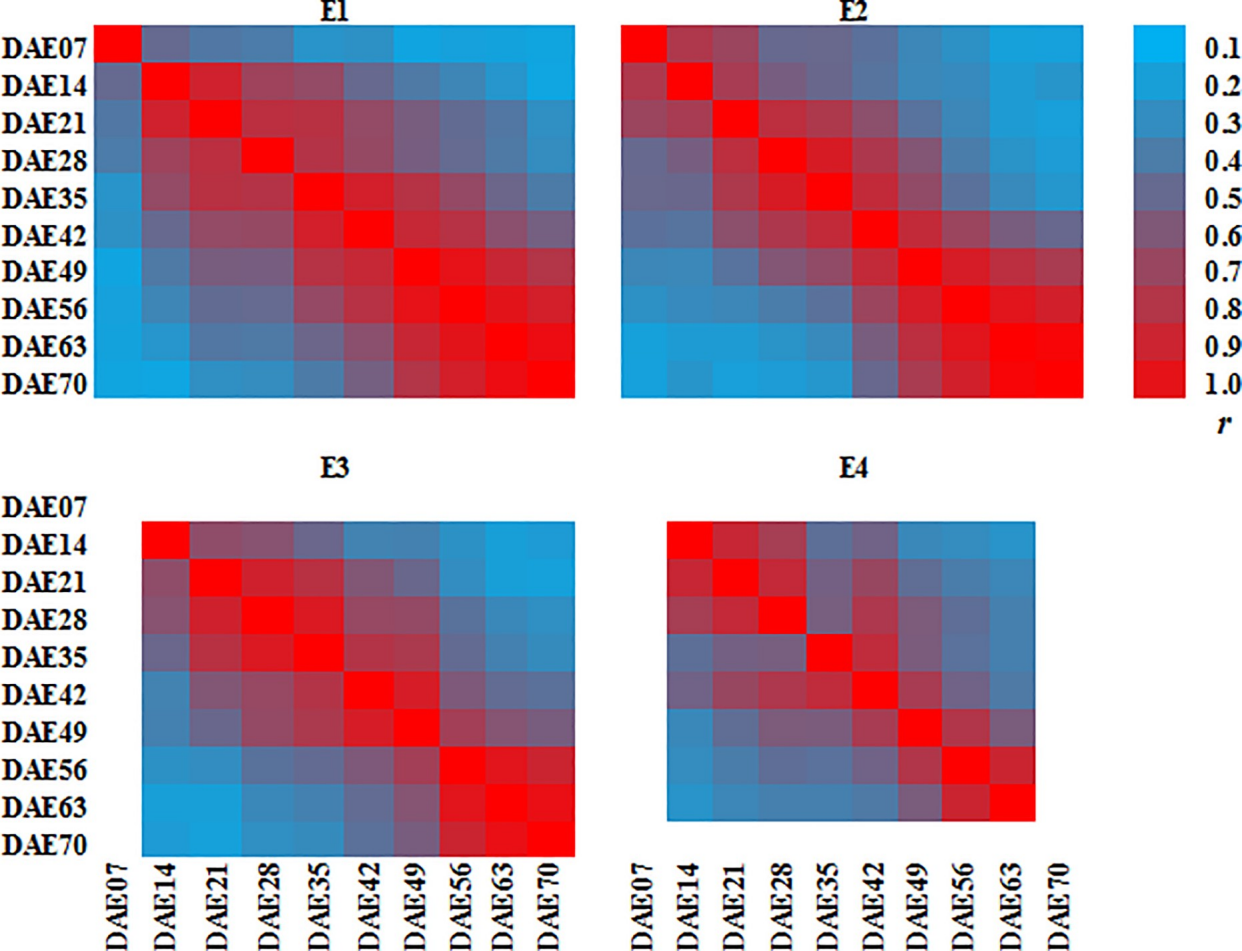
Phenotypic correlation coefficients among plant height at various stages. E1 represents the May 3, 2014 sowing date; E2 represents the May 25, 2014 sowing date; E3 represents the May 9, 2015 sowing date; E4 represents the May 25, 2015 sowing date; DAE represents plant height at days after emerging. The color picture can be downloaded in dio 10.1371/journal.pone.0224897.

The exceedance of genetic variance and heritability of unconditional variables over conditional ones suggested that variations of PH at some stages were increased by the prior stage (Table 1). Additionally, a positive correlation existed between PH at various stages and the values of two connected stages were higher than those between growing intervals, which indicated that PH at the (*t−*1)^th^ stage will enhance that at *t*^th^ stages (Fig 2).

The widespread variation among the various stages showed that it is necessary to explore the genetic basis of PH throughout the entire growth period. Furthermore, the lower conditional variance and tight correlations between two adjacent stages indicated that we should explain heredity in terms of accumulated variation from the initial to *t*^th^ stage and net variation from (*t*−1)^th^ to *t*^th^ stage.

### QTL analysis

**Fig 3:**
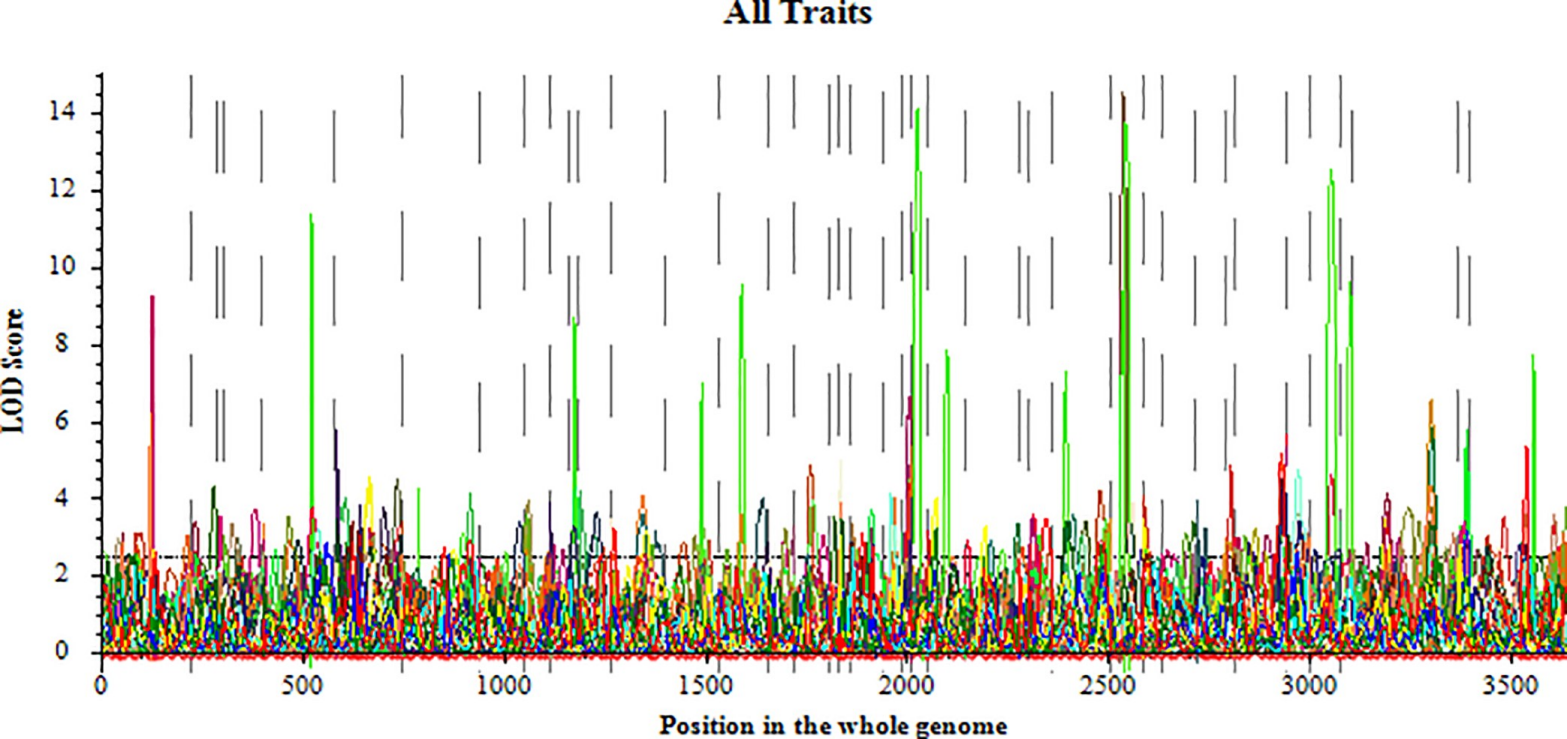
QTL for plant height on 20 chromosomes in the four-way recombinant inbred lines. The color picture can be downloaded in dio 10.1371/journal.pone.0224897.

A total of 161 QTL underlying PH with LOD over 2.5 (Fig 3) were identified at 10 stages in 95 regions on all 20 linkage groups (LGs) (S2 Table, S2 Fig).

**Fig 4:**
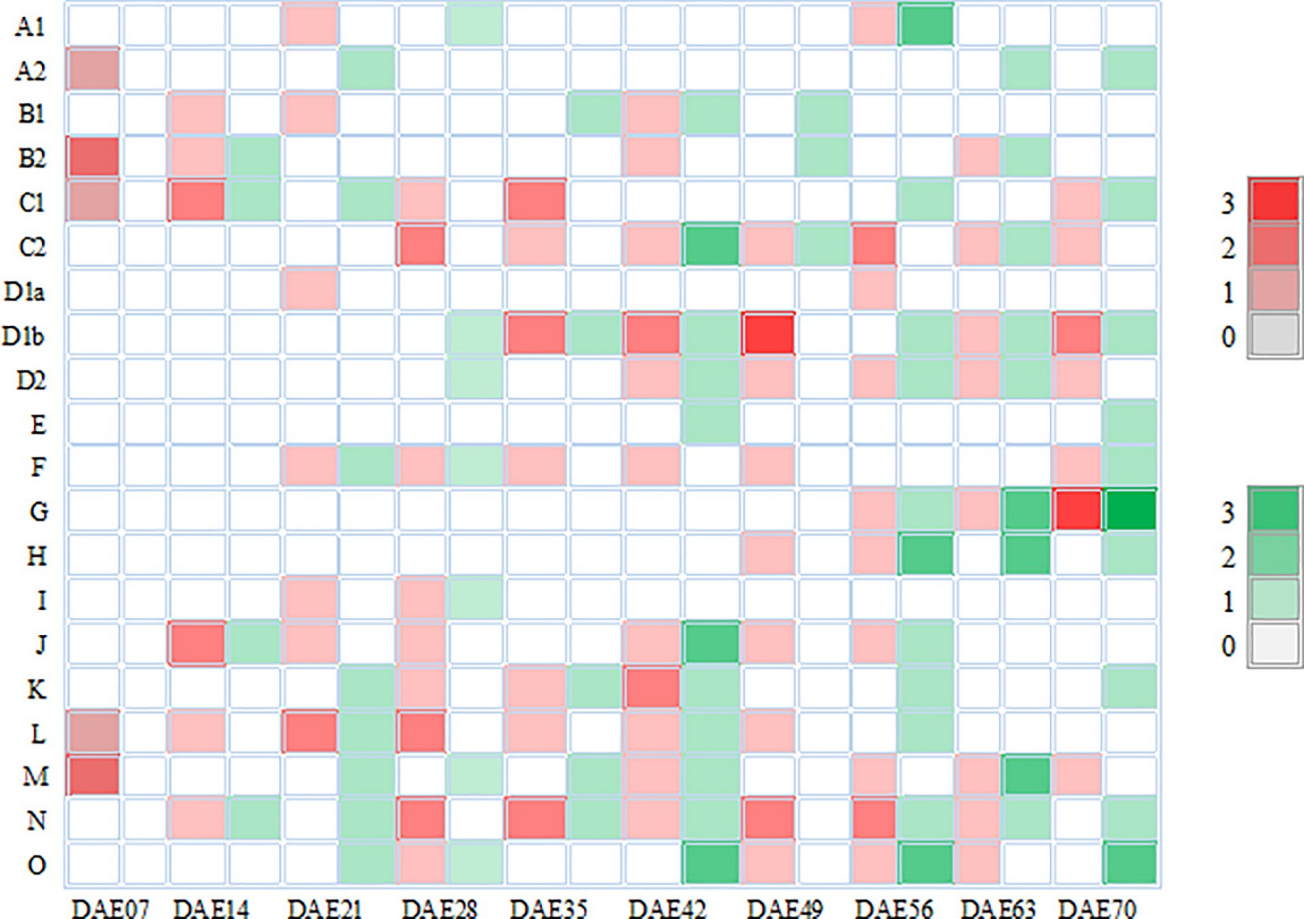
Frequency of QTL for plant height in 20 linkage groups. The color picture can be downloaded in dio 10.1371/journal.pone.0224897.

**Fig 5:**
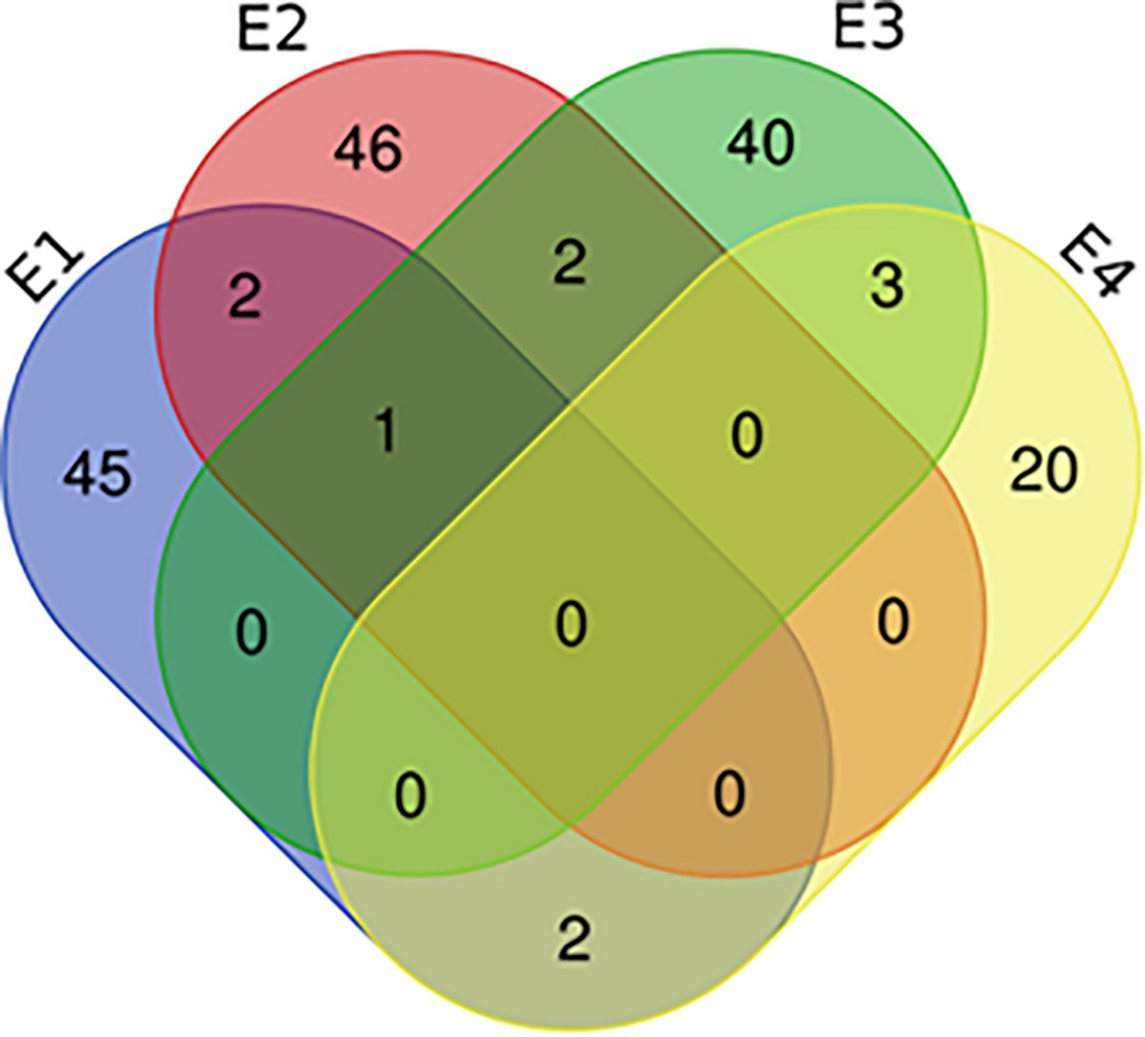
Frequency of QTL in the four environments. E1 represents the May 3, 2014 sowing date; E2 represents the May 25, 2014 sowing date; E3 represents the May 9, 2015 sowing date; E4 represents the May 25, 2015 sowing date; The color picture can be downloaded in dio 10.1371/journal.pone.0224897.

Among all QTL for PH, 89 were detected only by UVM in 52 regions, which indicated that the expression of these QTL functioned in PH continuously from emergence. Sixty-six QTL in 57 intervals were identified individually by CVM, which showed that these QTL only played a role during a specific stage and were completely influenced by adjacent stages. The remaining 9 QTL were simultaneously identified by UVM and CVM, which indicated that these QTL exhibited effects in some stages under partial influence of the previous stage. As the plants developed, the number of QTL increased gradually (Fig 4). The number reached the maximum at stage DAE56, and then decreased. There were 50, 51, 46, and 25 QTL detected in E1, E2, E3, and E4, respectively, and 10 were identified in multiple environments (Fig 5).

We detected 7, 8, 16, 19, 15, 27, 17, 27, 22, and 24 QTL for PH in the FW-RILs at ten stages of growth, respectively, with the ratio of genotypic variance explained by a single QTL of 5.44–14.56%, 2.42–12.73%, 3.43–12.76%, 3.01–18.68%, 1.17–15.57%, 3.13–13.01%, 3.07–12.08%, 1.79–14.82%, 2.82–16.57%, and 1.10–12.11%, respectively. There were 2, 2, 3, 3, 2, 3, 5, 4, 5, and 1 QTL with over 10% variance explained (PVE) in the ten stages, respectively. This indicated that PH is controlled by major-effect QTL (PVE > 10%) and minor-effect QTL (PVE ≤ 10%), and that the number of major-effect QTL was lower than that of minor-effect QTL. Among the 30 major-effect QTL, only one major-effect QTL (qPH70-G-1) was detected at the final stages, while the other 29 were identified throughout the growth period.

**Fig 6:**
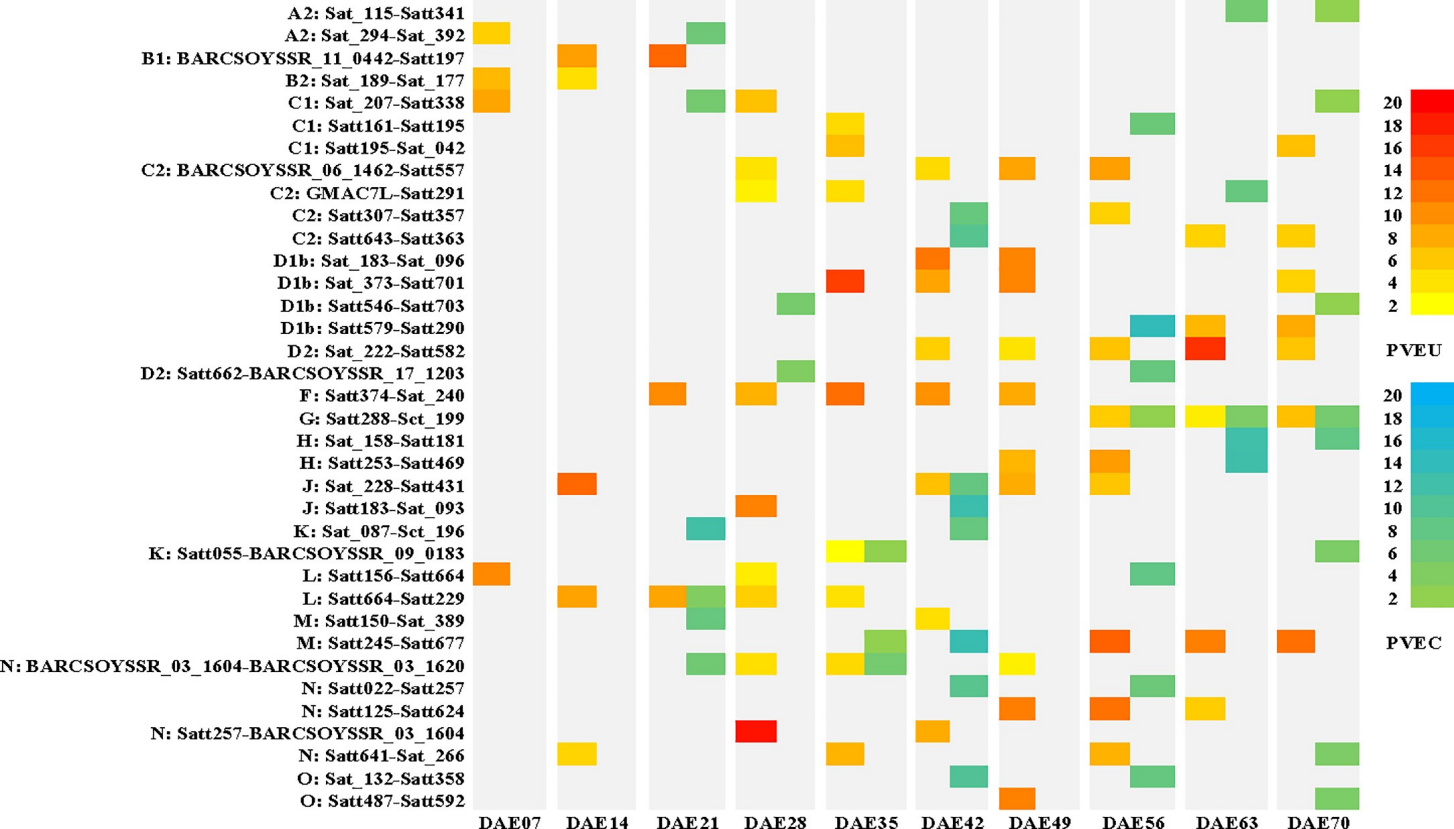
Temporal phenotypic variation explained ratio of QTL function on PH at multiple stages. PVEU: phenotypic variation explanation ratio of unconditional QTL; PVEC: phenotypic variation explanation ratio of conditional QTL; DAE *x*: *x* days after emergence; The color picture can be downloaded in dio 10.1371/journal.pone.0224897.

A total of 36 QTL controlling PH were detected in multiple stages (two to five stages; Fig 6). The huge difference of PVE among the various stages indicated that these genomic regions substantially influenced phenotypic variation during development. The region Sat_373-Satt701 in LG D1b contributed substantial phenotypic variation at DAE35, DAE42, DAE49, and DAE70. The interval Satt374-Sat_240 in LG F controlled PH continuously from DAE21 to DAE49. The QTL in Sat_228-Satt431 of LG J explained significant phenotypic variation at DAE14, DAE42, DAE49, and DAE56. QTL were detected in interval Satt245-Satt677 of LG M at five stages and showed PVE more than 10% at DAE42, DAE56, DAE63, and DAE70. In the interval of BARCSOYSSR_11_0442-Satt197 in LG B1 and Satt257-BARCSOYSSR_03_1604 in LG N two QTL were identified and played a role at DAE21 and DAE28 with PVE of over 10%. Two QTL from interval Sat_087-Sct_196 in LG K and Satt579-Satt290 in LG D1b expressed net additive effects at DAE21 and DAE56, respectively.

## Discussion

### Developmental analysis uncovered the genetic basis of PH over the whole growth period and enhanced the detection of QTL

Many QTL for PH have been identified based on phenotypic values at the harvest stage [7, 9–11,16–18,56–59]. However, the development of morphological traits is determined by the actions and interactions of many genes that are expressed differently during the various growth stages [23], and QTL mapping using phenotypic data measured at the mature stage cannot reveal all of the genes involved in the target quantitative traits throughout development [60–62]. It is necessary to conduct a genetic analysis over the entire developmental period, which not only gives a deeper insight into the genetic mechanisms behind the growth of the plant, but can also increase the detection of QTL underlying PH for practical marker-assisted breeding. In this study, we detected only eight unconditional QTL associated with PH in the final growth period, but by examining the entire developmental period, we identified a total of 89 unconditional QTL that are associated with PH. Similarly, in another study, Sun et al (2006) detected seven QTL for PH at the last stage (80DAE) and 28 throughout the entire growth period [8]. Therefore, it is important to detect QTL controlling PH throughout the whole growth period.

### Differences and advantages of unconditional and conditional QTL mapping

During the development of PH in soybean, which starts at emergence and ends at the seed filling stage, some genes act solely during a specific stage, but most genes function coordinately over a long period resulting in a tight correlation between adjacent stages (Fig 2). In order to evaluate the genetic role of the above genes, Zhu [24] proposed UVM to reflect cumulative genetic effects (from the initial stage to some other stages), and the CVM to reveal the net gene expression that occurred in a specific period of plant growth (between two adjacent stages) by excluding the influence of the previous growth stage. Generally, unconditional QTL can be used in marker-assisted breeding with consideration of the interaction of multiple genes over a longer developmental period, while conditional QTL are applied to find a major gene that expresses at specific stage.

There are two advantages of conditional QTL analysis. First, conditional QTL analysis could increase the number of identified QTL. In this research, 66 QTL associated with PH were detected only by conditional variable methods. Some QTL controlling PH, seed size, and linolenoyl acid content during soybean development were found using this method [8,20,40]. Secondly, conditional analysis could correct the estimation of additive effects and confirm the authenticity of identified QTL. In this study, nine QTL were found by unconditional and conditional methods, and the net additive effects were estimated after excluding the influence of the previous growth stage. At the same time, the repetitive detection supported the utility of these QTL.

### Use of FW-RILs increases the efficiency of QTL detection

Previous QTL mapping studies were mainly based on bi-parental crosses. Xu et al. (1996) proposed that new QTL could be discovered by increasing the number of parents in mapping studies [43]. Hayashi *et al*. noted that RILs resulting from a FWC would allow us to study the relationships between four parental alleles, and are therefore more useful in improving the efficiency of QTL detection than bi-parental RILs [46]. In addition, the population derived from a FWC can reflect the genetic diversity present in more parental lines, so more QTL can be detected from a FWC than a bi-parental cross [44–45, 48]. For example, qPH21-I-1 showed almost equal additive effects between allelic genotypes carried by Kenfeng14 and Heinong 48, Kenfeng 15 and Kenfeng 19 for DAE21 in E3, which indicates that this QTL would not have been detected based on bi-parental RIL populations derived from a cross between Kenfeng14 × Heinong 48 or Kenfeng 15 × Kenfeng 19 because there is not enough variation in allelic effect between two parents in these parental combinations. Similarly, the similarity of additive effects between allelic genotypes carried by Heinong 48 and Kenfeng 19 of qPH21-N-1 for DAE21|14 in E3 also showed it is impossible to detect this QTL based on RILs derived from Heinong 48 × Kenfeng 19. In this study, we used a FWC to investigate the conditional and unconditional QTL involved in PH in soybean, and to clarify the gene expression characteristics of PH in soybean in different growth periods.

### Genetic basis for the development of PH

By anchoring QTL found in previous and present studies in a public map [63], we analyzed the novelty and existence of the identified QTL (S3 Fig). We found that 76 genomic regions associated with PH detected in the present research had been identified in previous studies, while the remaining 19 regions were newly discovered in our study. For 30 major-effect QTL, 23 intervals containing QTL had been detected in previous studies and the remaining seven QTL were independent from the identified ones. The longer interval of qPH28-A1-1, qPH49-B1-1, qPH49-C2-2, qPH56-D1b-1, qPH49-D1b-1, qPH70-G-1, qPH63-H-2, qPH56-H-1, qPH28-I-1, qPH14-J-1, qPH21-K-1, qPH56-K-1, qPH49-L-1, qPH07-L-1, qPH56-L-1, qPH21-M-1, qPH42-M-2, qPH63-M-3, qPH07-M-1, qPH35-N-1, qPH63-N-2, qPH21-O-1, and qPH70-G-1 contained multiple QTL detected in the present and previous studies, and their contribution to genetic variation may be the sum of multiple genes. In the shorter region of qPH49-B2-1, qPH35-D1b-2, qPH63-H-3, qPH28-I-1, qPH42-J-2, qPH14-J-2, qPH28-O-1, and qPH42-O-1 only one QTL could be found, which functioned independently on PH. qPH63-C2-1 and qPH49-C2-2 are located in two adjacent regions with similar PVEs, which indicated that these two QTL may be one new QTL. Furthermore, qPH49-B2-1, qPH35-D1b-2, qPH63-H-3, qPH42-J-2, qPH28-O-1, qPH42-O-1, and qPH63-C2-1 controlled PH at other stages, and were first identified in this study. Therefore, these seven QTL may be valuable in gene cloning and molecular breeding for improving plant architecture in soybean.

## Conclusion

In this study, the progeny of a FWC population was used to assess the PH in soybean throughout the entire developmental period in four environments (sowing dates) by unconditional and conditional QTL mapping methods. As many as 85 QTL associated with PH were detected. Among them, 65 QTL associated with PH were confirmed for the facticity. Theoretically, our research constructed a temporal and spatial dynamic expression mode, which controlled the development of PH.

## Supporting information

S1 Fig Frequency distributions of plant height under two sowing period across two environments in a four-way cross (Kenfeng14 × Kenfeng15) × (Heinong48 × Kenfeng19). (DOCX)

S2 Fig Genetic maps of chromosomes showing QTL for the dynamic quantitative trait loci for plant height in soybean with a four-way recombinant inbreed lines. (DOCX)

S3 Fig QTL for plant height detected in present research and previous reports. (DOCX)

S1 Table Comparison of basic characters among four parents. (DOCX)

S2 Table QTL for PH at 10 stages detected under two sowing dates in two years. (DOCX)

## Abbreviations

PH: plant height
LG: linkage group
QTL: quantitative trait locus (or loci)
FW-RIL: four-way recombinant inbred lines
DAE: days after emergence
FWC: four-way cross
E1: the first sowing period in 2014
E2: the second sowing period in 2014
E3: the first sowing period in 2015
E4: the second sowing period in 2015
DAE*x*: *x* days after emergence
PCR: polymerase chain reaction
IM: interval mapping method
QAEE: quantity of additive effects expression
