# Supplementary material for: Mapping developmental QTL for plant height in soybean [Glycine max (L.) Merr.] using a four-way recombinant inbred line population
Source: PLoS One. 2019 Nov 20;14(11):e0224897. doi: 10.1371/journal.pone.0224897 (PMC6867651; doi:10.1371/journal.pone.0224897)
Supplement: S1 Fig — (DOCX) [file pone.0224897.s001.docx]

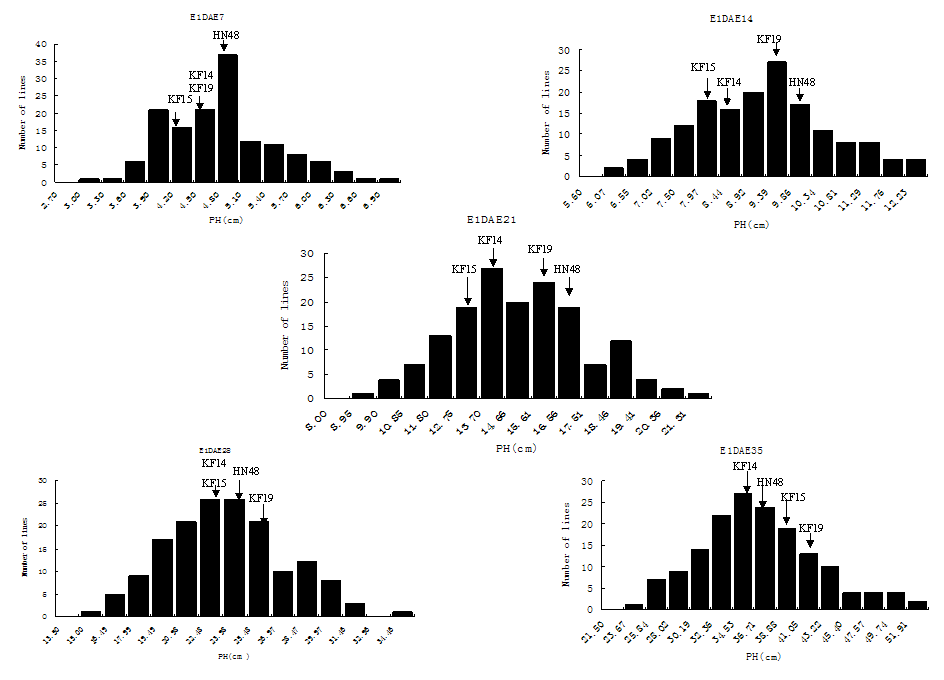

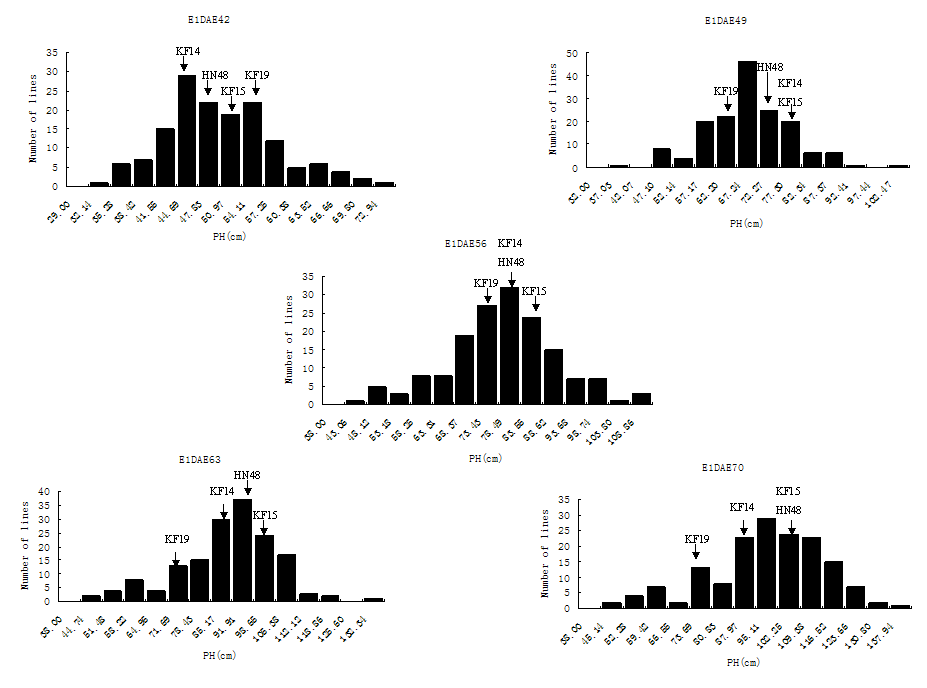

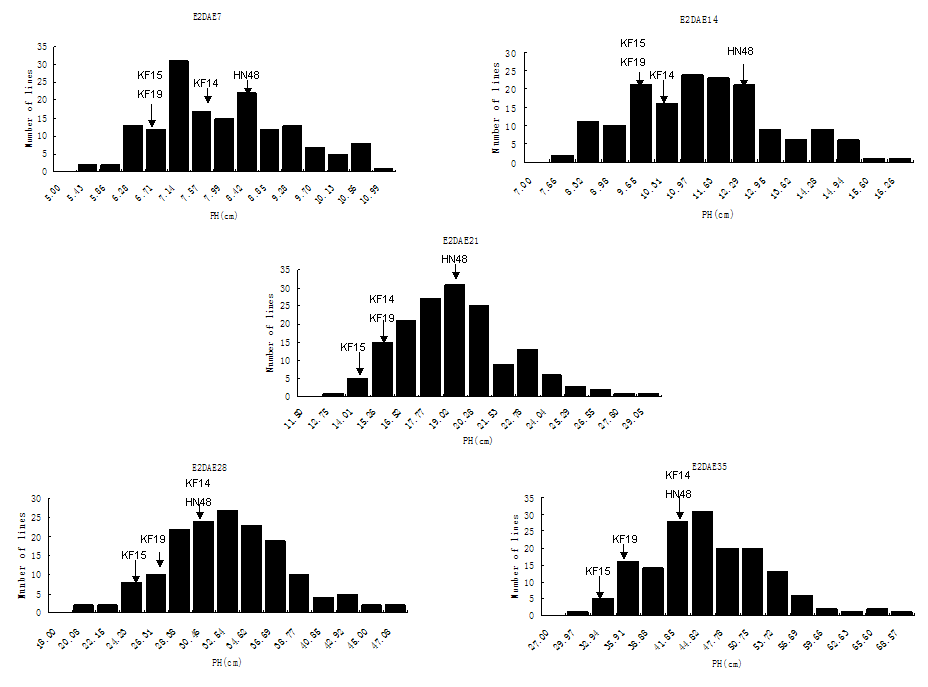

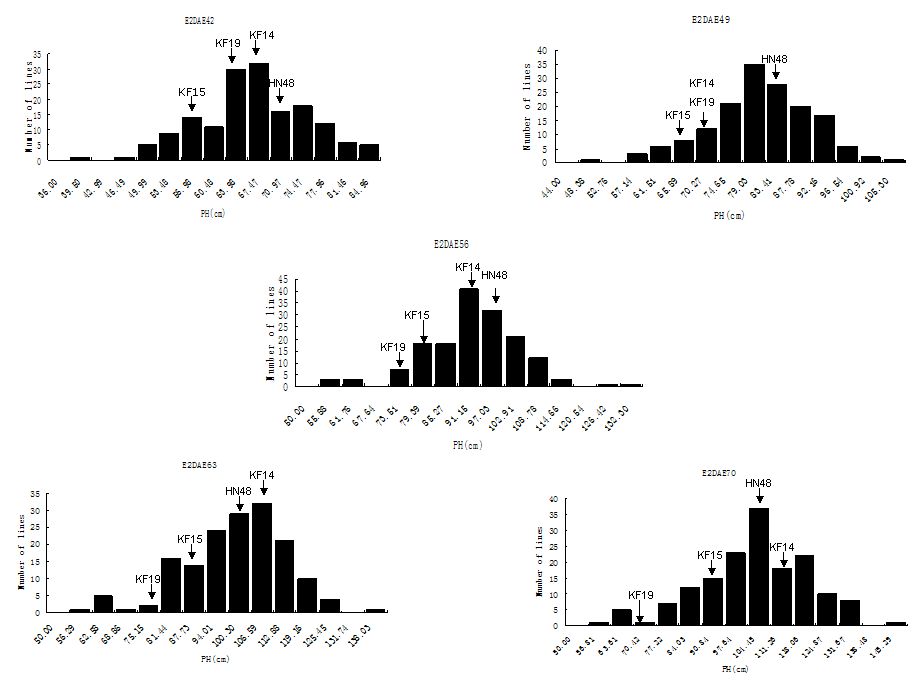

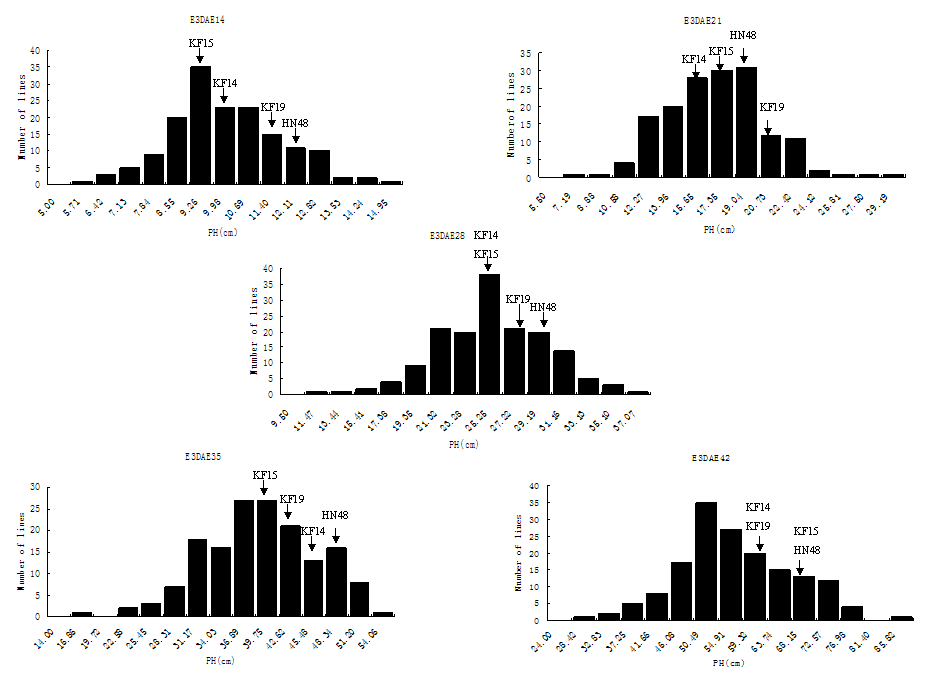

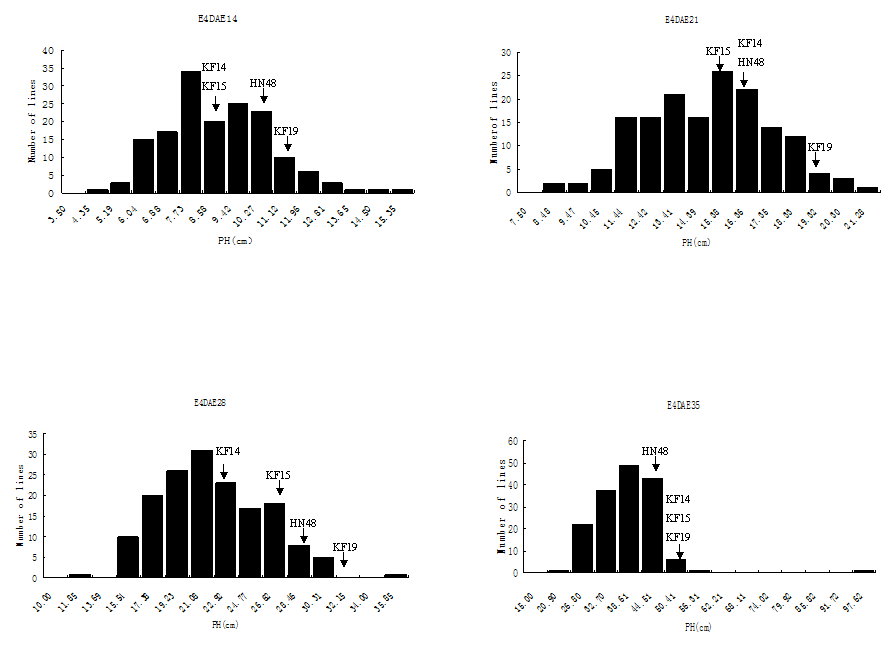

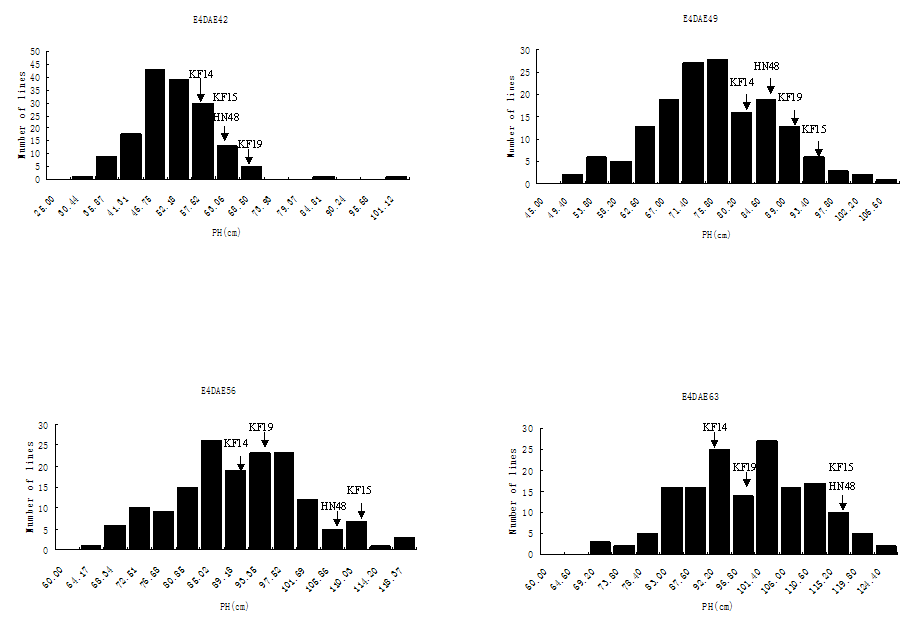


**S1 Fig.** Frequency distributions of plant height under two sowing period across two environments in a four-way cross (Kenfeng14×Kenfeng15) × (Heinong48×Kenfeng19)

^1^ PH: plant height; ^2^ KF14: Kenfeng14; ^3^ KF15: Kenfeng15; ^4^ HN48: Heinong48; ^5^ KF19: Kenfeng19; ^6^ DAE: days after emerging; ^7^ E1: the first sowing period in 2014; ^8^ E2: the second sowing period in 2014; ^9^ E3: the first sowing period in 2015; ^10^ E4: the second sowing period in 2015.
