# Supplementary material for: Mapping developmental QTL for plant height in soybean [Glycine max (L.) Merr.] using a four-way recombinant inbred line population
Source: PLoS One. 2019 Nov 20;14(11):e0224897. doi: 10.1371/journal.pone.0224897 (PMC6867651; doi:10.1371/journal.pone.0224897)
Supplement: S2 Fig — (DOCX) [file pone.0224897.s002.docx]

**S2 Fig. Genetic maps of chromosomes showing QTL for the dynamic quantitative trait loci for plant height in soybean with a four-way recombinant inbreed lines**

^1^ The words in parentheses showed the traits in some environments; ^2^ E1, the first sowing period in 2014; ^3^ E2, the second sowing period in 2014; ^4^ E3, the first sowing period in 2015; ^5^ E4, the second sowing period in 2015;^6^ DAE, days after emerge; DAEx|y: net increase during the time interval from days y to x after emergence.
