# Supplementary material for: Mapping developmental QTL for plant height in soybean [Glycine max (L.) Merr.] using a four-way recombinant inbred line population
Source: PLoS One. 2019 Nov 20;14(11):e0224897. doi: 10.1371/journal.pone.0224897 (PMC6867651; doi:10.1371/journal.pone.0224897)
Supplement: S3 Fig — (DOCX) [file pone.0224897.s003.docx]

**S3 Fig. QTL for plant height detected in present research and previous reports**

^1^ Red bars represent QTL detected in this research and green ones represent QTL from previous reports.
