# Supplementary material for: Mapping developmental QTL for plant height in soybean [Glycine max (L.) Merr.] using a four-way recombinant inbred line population
Source: PLoS One. 2019 Nov 20;14(11):e0224897. doi: 10.1371/journal.pone.0224897 (PMC6867651; doi:10.1371/journal.pone.0224897)
Supplement: S1 Table — (DOCX) [file pone.0224897.s004.docx]

**S1 Table. Source and plant height of four parents**

| Variety | Source | Plant height | Breeding institution |
| --- | --- | --- | --- |
| Kenfeng14 | Suinong10 × Changnong5 | 100cm | Heilongjiang Academy of Land-reclamation Sciences, Jiamusi, China |
| Kenfeng15 | Suinong14 × Kenjiao9307 | 85cm | Heilongjiang Academy of Land-reclamation Sciences, Jiamusi, China |
| Heinong48 | Ha90-6719 × Sui90-5888 | 88cm | Heilongjiang Academy of Agricultural Sciences, Harbin, China |
| Kenfeng19 | Hefeng25 × (Kenfeng4 ×Gong8861-0) | 65cm | Heilongjiang Academy of Land-reclamation Sciences, Jiamusi, China |
