# Supplementary material for: Mapping developmental QTL for plant height in soybean [Glycine max (L.) Merr.] using a four-way recombinant inbred line population
Source: PLoS One. 2019 Nov 20;14(11):e0224897. doi: 10.1371/journal.pone.0224897 (PMC6867651; doi:10.1371/journal.pone.0224897)
Supplement: S2 Table — (DOCX) [file pone.0224897.s005.docx]

**S2 Table. QTL for PH at 10 stages detected under two sowing dates in two years**

| QTL | Chromosome | Interval | Unconditional variable | | | | | | |  | Conditional variable | | | | | | |
| --- | --- | --- | --- | --- | --- | --- | --- | --- | --- | --- | --- | --- | --- | --- | --- | --- | --- |
|  |  |  | LOD | PVE(%) | Add1 | Add2 | Add3 | Add4 | Environments |  | LOD | PVE(%) | Add1 | Add2 | Add3 | Add4 | Environments |
| qPH07-A2-1 | A2 | Sat_294-Sat_392 | 3.01 | 5.44 | 0.51 | -0.09 | -0.60 | 0.18 | E2 |  |  |  |  |  |  |  |  |
| qPH07-B2-1 | B2 | Sat_189-Sat_177 | 3.66 | 6.95 | -0.28 | -0.06 | 0.43 | -0.09 | E1 |  |  |  |  |  |  |  |  |
| qPH07-B2-2 | B2 | Satt070-Sat_355 | 3.29 | 7.06 | -0.01 | -0.61 | 0.75 | -0.13 | E2 |  |  |  |  |  |  |  |  |
| qPH07-C1-1 | C1 | Sat_207-Satt338 | 4.09 | 8.41 | -0.37 | 0.40 | -0.07 | 0.04 | E1 |  |  |  |  |  |  |  |  |
| qPH07-L-1 | L | Satt156-Satt664 | 3.78 | 10.40 | -0.59 | -0.52 | 0.52 | 0.60 | E2 |  |  |  |  |  |  |  |  |
| qPH07-M-1 | M | Satt435-Sat_244 | 3.52 | 14.56 | -0.18 | -0.32 | 0.63 | -0.12 | E1 |  |  |  |  |  |  |  |  |
| qPH07-M-2 | M | Satt677-Satt728 | 4.38 | 6.89 | 0.34 | 0.36 | -0.05 | -0.66 | E2 |  |  |  |  |  |  |  |  |
| qPH14-B1-1 | B1 | BARCSOYSSR_11_0442-Satt197 | 3.43 | 8.78 | 0.39 | 0.08 | 0.26 | -0.72 | E1 |  |  |  |  |  |  |  |  |
| qPH14-B2-1 | B2 | Sat_189-Sat_177 | 3.22 | 4.33 | -0.31 | -0.52 | 1.06 | -0.24 | E4 |  |  |  |  |  |  |  |  |
| qPH14-C1-1 | C1 | Sat_042-Sat_207 | 3.18 | 2.42 | -0.45 | 1.02 | -0.58 | 0.01 | E3 |  |  |  |  |  |  |  |  |
| qPH14-C1-2 | C1 | Satt338-Satt164 | 4.20 | 9.56 | -0.60 | 0.43 | -0.34 | 0.52 | E1 |  |  |  |  |  |  |  |  |
| qPH14-J-1 | J | Sat_228-Satt431 | 4.24 | 12.73 | -0.33 | -0.42 | -0.93 | 1.68 | E4 |  |  |  |  |  |  |  |  |
| qPH14-J-2 | J | Satt529-Satt183 | 3.82 | 11.02 | -0.56 | -0.46 | 1.35 | -0.34 | E2 |  |  |  |  |  |  |  |  |
| qPH14-L-1 | L | Satt664-Satt229 | 4.48 | 8.50 | -0.60 | -0.67 | 0.10 | 1.17 | E2 |  |  |  |  |  |  |  |  |
| qPH14-N-1 | N | Satt641-Sat_266 | 5.34 | 5.04 | -0.57 | -0.87 | 1.23 | 0.22 | E3 |  |  |  |  |  |  |  |  |
| qPH21-A1-1 | A1 | Satt684-Sat_137 | 3.26 | 7.08 | -1.24 | -1.10 | 1.27 | 1.07 | E3 |  |  |  |  |  |  |  |  |
| qPH21-A2-1 | A2 | Sat_294-Sat_392 |  |  |  |  |  |  |  |  | 3.61 | 6.55 | -0.46 | 0.20 | 0.96 | -0.71 | E1 |
| qPH21-B1-1 | B1 | BARCSOYSSR_11_0442-Satt197 | 3.07 | 12.76 | 0.18 | 0.07 | 0.90 | -1.16 | E1 |  |  |  |  |  |  |  |  |
| qPH21-C1-1 | C1 | Sat_207-Satt338 |  |  |  |  |  |  |  |  | 3.79 | 5.65 | 0.17 | 1.58 | 0.08 | -1.82 | E3 |
| qPH21-D1a-1 | D1a | Satt077-Satt198 | 3.10 | 9.44 | -0.43 | -0.92 | 0.83 | 0.52 | E4 |  |  |  |  |  |  |  |  |
| qPH21-F-1 | F | FlwC-Sat_039 |  |  |  |  |  |  |  |  | 3.49 | 3.43 | -0.82 | -0.37 | -0.35 | 1.54 | E3 |
| qPH21-F-2 | F | Satt374-Sat_240 | 3.00 | 10.14 | 0.20 | 0.35 | 0.60 | -1.14 | E1 |  |  |  |  |  |  |  |  |
| qPH21-I-1 | I | Sat_268-Satt270 | 3.06 | 4.46 | 0.92 | -0.91 | 0.92 | -0.92 | E3 |  |  |  |  |  |  |  |  |
| qPH21-J-1 | J | BARCSOYSSR_16_0500-BARCSOYSSR_16_0566 | 3.18 | 4.78 | -0.06 | -0.17 | -1.26 | 1.49 | E3 |  |  |  |  |  |  |  |  |
| qPH21-K-1 | K | Sat_087-Sct_196 |  |  |  |  |  |  |  |  | 3.58 | 11.76 | -0.43 | -0.76 | -0.35 | 1.54 | E1 |
| qPH21-L-1 | L | Satt664-Satt229 | 3.48 | 8.40 | -1.24 | -0.87 | 0.81 | 1.31 | E2 |  |  |  |  |  |  |  |  |
|  |  |  | 5.46 | 8.97 | -1.75 | -0.83 | 1.43 | 1.15 | E3 |  | 3.67 | 4.05 | -1.36 | -0.47 | 1.11 | 0.73 | E3 |
| qPH21-M-1 | M | Satt150-Sat_389 |  |  |  |  |  |  |  |  | 3.01 | 7.36 | -0.75 | -0.08 | 1.11 | -0.28 | E1 |
| qPH21-N-1 | N | BARCSOYSSR_03_1604-BARCSOYSSR_03_1620 |  |  |  |  |  |  |  |  | 4.18 | 6.10 | 0.59 | -2.06 | 0.77 | 0.71 | E3 |
| qPH21-O-1 | O | Satt345-Satt478 |  |  |  |  |  |  |  |  | 3.93 | 7.57 | 0.41 | -1.17 | 0.85 | -0.09 | E2 |
| qPH28-A1-1 | A1 | Satt200-Satt717 |  |  |  |  |  |  |  |  | 3.53 | 8.18 | 0.20 | 1.70 | -1.26 | -0.64 | E1 |
| qPH28-C1-1 | C1 | Sat_207-Satt338 | 4.49 | 6.34 | -0.10 | 3.17 | -1.03 | -2.04 | E3 |  |  |  |  |  |  |  |  |
| qPH28-C2-1 | C2 | BARCSOYSSR_06_1462-Satt557 | 3.16 | 4.00 | -1.77 | -2.45 | 3.31 | 0.92 | E2 |  |  |  |  |  |  |  |  |
| qPH28-C2-2 | C2 | GMAC7L-Satt291 | 4.74 | 3.01 | 0.89 | 2.88 | -1.08 | -2.69 | E2 |  |  |  |  |  |  |  |  |
| qPH28-D1b-1 | D1b | Satt546-Satt703 |  |  |  |  |  |  |  |  | 3.76 | 5.33 | -0.60 | -0.73 | -0.08 | 1.41 | E4 |
| qPH28-D2-1 | D2 | Satt662-BARCSOYSSR_17_1203 |  |  |  |  |  |  |  |  | 3.04 | 4.00 | -1.04 | 0.30 | 0.90 | -0.16 | E4 |
| qPH28-F-1 | F | Sat_298-AW186493 |  |  |  |  |  |  |  |  | 3.32 | 4.08 | 0.23 | -0.78 | 1.08 | -0.54 | E1 |
| qPH28-F-2 | F | Satt374-Sat_240 | 3.62 | 7.39 | 0.27 | 0.75 | 0.79 | -1.81 | E1 |  |  |  |  |  |  |  |  |
| qPH28-I-1 | I | Satt354-Satt239 | 3.02 | 12.79 | -1.52 | -0.80 | 1.64 | 0.68 | E4 |  | 4.28 | 9.09 | -0.73 | 1.43 | -1.19 | 0.48 | E3 |
| qPH28-J-1 | J | Satt183-Sat_093 | 3.06 | 10.81 | -1.54 | 0.16 | 1.61 | -0.23 | E4 |  |  |  |  |  |  |  |  |
| qPH28-K-1 | K | Satt588-Satt196 | 3.00 | 6.06 | -0.39 | 1.72 | -0.27 | -1.06 | E1 |  |  |  |  |  |  |  |  |
| qPH28-L-1 | L | Satt156-Satt664 | 3.66 | 3.37 | -1.63 | -2.66 | 2.35 | 1.94 | E2 |  |  |  |  |  |  |  |  |
| qPH28-L-2 | L | Satt664-Satt229 | 5.06 | 5.46 | -2.20 | -0.98 | 1.21 | 1.97 | E3 |  |  |  |  |  |  |  |  |
| qPH28-M-1 | M | Sat_244-Satt245 |  |  |  |  |  |  |  |  | 3.78 | 7.50 | -0.47 | -0.13 | -0.81 | 1.41 | E3 |
| qPH28-N-1 | N | BARCSOYSSR_03_1604-BARCSOYSSR_03_1620 | 3.45 | 4.32 | -0.75 | -1.91 | 2.05 | 0.60 | E3 |  |  |  |  |  |  |  |  |
| qPH28-N-2 | N | Satt257-BARCSOYSSR_03_1604 | 4.46 | 18.68 | -0.49 | -0.28 | 2.81 | -2.04 | E1 |  |  |  |  |  |  |  |  |
| qPH28-O-1 | O | BARCSOYSSR_10_0581-Sat_291 |  |  |  |  |  |  |  |  | 4.21 | 8.75 | 1.22 | -0.95 | -1.20 | 0.93 | E1 |
| qPH28-O-2 | O | Satt581-Sat_108 | 3.22 | 6.80 | -1.50 | -0.45 | 1.03 | 0.92 | E1 |  |  |  |  |  |  |  |  |
| qPH35-B1-1 | B1 | Sat_123-Satt359 |  |  |  |  |  |  |  |  | 3.00 | 4.36 | -0.74 | -0.92 | 1.97 | -0.31 | E1 |
| qPH35-C1-1 | C1 | Satt161-Satt195 | 3.62 | 4.67 | 0.39 | 2.88 | 0.99 | -4.26 | E3 |  |  |  |  |  |  |  |  |
| qPH35-C1-2 | C1 | Satt195-Sat_042 | 3.01 | 6.63 | 0.28 | -0.23 | 2.72 | -2.77 | E1 |  |  |  |  |  |  |  |  |
| qPH35-C2-1 | C2 | GMAC7L-Satt291 | 3.02 | 4.39 | 0.40 | 4.32 | -0.66 | -4.06 | E2 |  |  |  |  |  |  |  |  |
| qPH35-D1a-1 | D1b | Sat_069-Sat_183 | 3.39 | 3.61 | -2.10 | -1.98 | 1.00 | 3.08 | E3 |  |  |  |  |  |  |  |  |
| qPH35-D1b-1 | D1b | Sat_373-Satt701 | 3.32 | 15.57 | 3.69 | -2.36 | 0.84 | -2.17 | E1 |  |  |  |  |  |  |  |  |
| qPH35-D1b-2 | D1b | Satt703-Sat_069 |  |  |  |  |  |  |  |  | 3.68 | 7.64 | -0.82 | -1.49 | 1.81 | 0.50 | E3 |
| qPH35-F-1 | F | Satt374-Sat_240 | 4.41 | 12.27 | 1.19 | -1.49 | 2.81 | -2.51 | E1 |  |  |  |  |  |  |  |  |
| qPH35-K-1 | K | Satt055-BARCSOYSSR_09_0183 | 8.62 | 1.17 | -14.04 | -15.53 | 45.63 | -16.06 | E4 |  | 14.37 | 1.29 | -14.51 | -15.12 | 45.73 | -16.10 | E4 |
| qPH35-L-1 | L | Satt664-Satt229 | 4.11 | 4.13 | -3.03 | -1.35 | 2.67 | 1.71 | E3 |  |  |  |  |  |  |  |  |
| qPH35-M-1 | M | Satt245-Satt677 |  |  |  |  |  |  |  |  | 12.47 | 1.28 | -15.52 | 45.43 | -14.10 | -15.80 | E4 |
| qPH35-N-1 | N | BARCSOYSSR_03_1604-BARCSOYSSR_03_1620 | 5.19 | 4.69 | 1.07 | -3.90 | 2.60 | 0.23 | E3 |  | 3.39 | 5.52 | 1.43 | -1.56 | 0.24 | -0.11 | E3 |
| qPH35-N-2 | N | Satt641-Sat_266 | 3.60 | 7.24 | -1.74 | -2.36 | 4.66 | -0.55 | E3 |  |  |  |  |  |  |  |  |
| qPH42-B1-1 | B1 | BARCSOYSSR_11_1481-Sat_331 |  |  |  |  |  |  |  |  | 3.50 | 3.86 | -1.82 | -1.36 | -1.23 | 4.41 | E3 |
| qPH42-B1-2 | B1 | Satt197-Sat_247 | 4.08 | 7.69 | -1.35 | 2.48 | 2.53 | -3.66 | E1 |  |  |  |  |  |  |  |  |
| qPH42-B2-1 | B2 | Sat_182-Satt168 | 3.05 | 3.13 | 3.17 | 1.95 | -3.21 | -1.90 | E2 |  |  |  |  |  |  |  |  |
| qPH42-C2-1 | C2 | BARCSOYSSR_06_1462-Satt557 | 3.36 | 4.64 | -2.69 | -2.36 | 3.73 | 1.32 | E2 |  |  |  |  |  |  |  |  |
| qPH42-C2-2 | C2 | Satt307-Satt357 |  |  |  |  |  |  |  |  | 3.21 | 7.28 | -0.57 | -2.03 | -0.48 | 3.07 | E1 |
| qPH42-C2-3 | C2 | Satt643-Satt363 |  |  |  |  |  |  |  |  | 3.57 | 9.31 | 1.13 | -2.14 | -1.46 | 2.47 | E4 |
| qPH42-D1b-1 | D1b | Sat_183-Sat_096 | 3.90 | 11.62 | -3.90 | -4.53 | 1.74 | 6.70 | E3 |  |  |  |  |  |  |  |  |
| qPH42-D1b-2 | D1b | Sat_373-Satt701 | 3.10 | 8.47 | 4.19 | -3.19 | 1.19 | -2.19 | E1 |  |  |  |  |  |  |  |  |
| qPH42-D1b-3 | D1b | Satt558-BARCSOYSSR_02_0607 |  |  |  |  |  |  |  |  | 3.71 | 7.06 | 0.82 | 1.62 | -2.31 | -0.14 | E1 |
| qPH42-D2-1 | D2 | Sat_222-Satt582 | 3.38 | 5.41 | -1.92 | -2.27 | 5.50 | -1.31 | E3 |  |  |  |  |  |  |  |  |
| qPH42-D2-2 | D2 | Satt582-Satt002 |  |  |  |  |  |  |  |  | 3.04 | 7.55 | -1.77 | 2.77 | -0.54 | -0.45 | E1 |
| qPH42-E-1 | E | Satt491-Satt699 |  |  |  |  |  |  |  |  | 3.09 | 6.64 | -3.30 | 0.20 | 1.32 | 1.78 | E2 |
| qPH42-F-1 | F | Satt374-Sat_240 | 5.21 | 9.70 | 1.41 | -1.38 | 3.86 | -3.89 | E1 |  |  |  |  |  |  |  |  |
| qPH42-J-1 | J | Sat_228-Satt431 | 4.20 | 6.46 | -2.84 | 2.27 | -1.88 | 2.45 | E1 |  | 4.53 | 7.63 | -1.99 | 3.66 | -1.84 | 0.17 | E2 |
| qPH42-J-2 | J | Satt183-Sat_093 |  |  |  |  |  |  |  |  | 4.01 | 12.56 | -3.13 | -0.85 | 2.13 | 1.86 | E4 |
| qPH42-K-1 | K | Sat_087-Sct_196 |  |  |  |  |  |  |  |  | 3.19 | 7.39 | -2.62 | -0.16 | 0.25 | 2.52 | E2 |
| qPH42-K-2 | K | Satt247-Satt727 | 4.56 | 6.69 | 3.41 | -3.97 | -2.41 | 2.98 | E2 |  |  |  |  |  |  |  |  |
| qPH42-K-3 | K | Satt349-BARCSOYSSR_09_0849 | 3.32 | 7.99 | 3.13 | -0.84 | 1.82 | -4.12 | E1 |  |  |  |  |  |  |  |  |
| qPH42-L-1 | L | Satt076-Satt156 |  |  |  |  |  |  |  |  | 3.21 | 6.82 | -2.41 | 0.58 | 1.88 | -0.06 | E4 |
| qPH42-L-2 | L | Satt182-Sat_301 | 6.61 | 7.21 | 5.35 | 0.90 | -3.18 | -3.08 | E2 |  |  |  |  |  |  |  |  |
| qPH42-M-1 | M | Satt150-Sat_389 | 4.30 | 4.29 | -3.96 | -0.97 | 2.82 | 2.11 | E2 |  |  |  |  |  |  |  |  |
| qPH42-M-2 | M | Satt245-Satt677 |  |  |  |  |  |  |  |  | 3.08 | 13.01 | 2.07 | -6.29 | 1.81 | 2.40 | E2 |
| qPH42-N-1 | N | Satt022-Satt257 |  |  |  |  |  |  |  |  | 3.48 | 9.06 | -0.49 | -0.84 | 3.02 | -1.69 | E1 |
| qPH42-N-2 | N | Satt257-BARCSOYSSR_03_1604 | 3.53 | 7.90 | 0.56 | -1.26 | 4.14 | -3.43 | E1 |  |  |  |  |  |  |  |  |
| qPH42-O-1 | O | Sat_132-Satt358 |  |  |  |  |  |  |  |  | 3.74 | 9.92 | -3.25 | -3.11 | 8.11 | -1.75 | E3 |
| qPH42-O-2 | O | Satt478-Satt188 |  |  |  |  |  |  |  |  | 3.37 | 5.93 | -2.20 | 1.39 | -0.62 | 1.44 | E2 |
| qPH49-B1-1 | B1 | Satt453-BARCSOYSSR_11_0442 |  |  |  |  |  |  |  |  | 3.13 | 6.84 | -1.32 | -0.35 | 4.04 | -2.36 | E2 |
| qPH49-B2-1 | B2 | Sat_355-Sat_189 |  |  |  |  |  |  |  |  | 3.57 | 4.78 | 1.63 | -2.06 | 2.45 | -2.02 | E2 |
| qPH49-C2-1 | C2 | BARCSOYSSR_06_1462-Satt557 | 3.53 | 8.49 | -2.00 | -3.23 | 3.88 | 1.35 | E2 |  |  |  |  |  |  |  |  |
| qPH49-C2-2 | C2 | Satt291-Satt281 |  |  |  |  |  |  |  |  | 3.14 | 3.27 | 0.63 | 1.65 | 0.47 | -2.76 | E2 |
| qPH49-D1b-1 | D1b | Sat_183-Sat_096 | 6.18 | 11.35 | -1.77 | -7.04 | 1.66 | 7.15 | E3 |  |  |  |  |  |  |  |  |
|  | D1b | Sat_183-Sat_096 | 3.04 | 8.96 | -0.22 | -1.70 | 6.99 | -5.07 | E4 |  |  |  |  |  |  |  |  |
| qPH49-D1b-2 | D1b | Sat_289-Satt271 | 3.44 | 5.48 | 3.87 | 1.30 | -0.42 | -4.75 | E4 |  |  |  |  |  |  |  |  |
| qPH49-D1b-3 | D1b | Sat_373-Satt701 | 3.15 | 10.73 | 6.31 | -2.90 | 0.06 | -3.47 | E1 |  |  |  |  |  |  |  |  |
| qPH49-D2-1 | D2 | Sat_222-Satt582 | 4.08 | 4.02 | -2.11 | -1.80 | 5.68 | -1.77 | E3 |  |  |  |  |  |  |  |  |
| qPH49-F-1 | F | Satt374-Sat_240 | 3.64 | 8.07 | 2.22 | -3.15 | 4.28 | -3.35 | E1 |  |  |  |  |  |  |  |  |
| qPH49-H-1 | H | Satt253-Satt469 | 3.33 | 7.17 | 0.95 | 1.54 | -5.88 | 3.38 | E4 |  |  |  |  |  |  |  |  |
| qPH49-J-1 | J | Sat_228-Satt431 | 5.63 | 6.56 | -2.70 | 2.67 | 5.45 | -5.42 | E3 |  |  |  |  |  |  |  |  |
|  | J | Sat_228-Satt431 | 5.92 | 9.29 | -5.65 | 2.85 | -0.95 | 3.75 | E4 |  |  |  |  |  |  |  |  |
| qPH49-L-1 | L | BARCSOYSSR_19_0466-Satt313 | 4.10 | 12.80 | 4.33 | -4.41 | 1.36 | -1.28 | E2 |  |  |  |  |  |  |  |  |
| qPH49-N-1 | N | BARCSOYSSR_03_1604-BARCSOYSSR_03_1620 | 3.02 | 3.07 | 0.37 | -4.60 | 3.10 | 1.13 | E3 |  |  |  |  |  |  |  |  |
| qPH49-N-2 | N | Satt125-Satt624 | 3.70 | 11.13 | 4.57 | 0.62 | -4.99 | -0.20 | E2 |  |  |  |  |  |  |  |  |
| qPH49-O-1 | O | Satt487-Satt592 | 4.39 | 10.93 | 3.95 | -3.61 | -5.04 | 4.70 | E4 |  |  |  |  |  |  |  |  |
| qPH56-A1-1 | A1 | AZ536570-BARCSOYSSR_05_0513 |  |  |  |  |  |  |  |  | 3.28 | 4.87 | 1.52 | -1.84 | -1.24 | 1.56 | E1 |
| qPH56-A1-2 | A1 | Sat_410-Satt593 | 3.63 | 5.20 | -1.78 | 5.10 | -5.09 | 1.78 | E2 |  | 3.51 | 4.43 | -0.39 | 2.50 | -3.08 | 0.97 | E2 |
| qPH56-C1-1 | C1 | Satt161-Satt195 |  |  |  |  |  |  |  |  | 3.50 | 6.70 | -1.41 | 0.06 | -2.53 | 3.89 | E2 |
| qPH56-C2-1 | C2 | BARCSOYSSR_06_1462-Satt557 | 6.11 | 8.72 | -3.59 | -5.18 | 4.42 | 4.35 | E2 |  |  |  |  |  |  |  |  |
| qPH56-C2-2 | C2 | Satt307-Satt357 | 3.10 | 5.28 | 2.84 | -1.17 | 4.76 | -6.43 | E2 |  |  |  |  |  |  |  |  |
| qPH56-D1a-1 | D1a | Satt580-AZ302047 | 3.01 | 4.42 | -2.98 | 0.43 | -4.59 | 7.14 | E1 |  |  |  |  |  |  |  |  |
| qPH56-D1b-1 | D1b | Satt579-Satt290 |  |  |  |  |  |  |  |  | 5.42 | 13.82 | 2.19 | -3.98 | -0.58 | 2.37 | E1 |
| qPH56-D2-1 | D2 | Sat_222-Satt582 | 3.26 | 6.28 | -3.72 | -2.39 | 6.47 | -0.37 | E3 |  |  |  |  |  |  |  |  |
| qPH56-D2-2 | D2 | Satt662-BARCSOYSSR_17_1203 |  |  |  |  |  |  |  |  | 4.29 | 7.21 | 2.62 | 0.97 | -2.09 | -1.50 | E1 |
| qPH56-G-1 | G | Satt288-Sct_199 | 3.07 | 5.66 | -0.43 | 5.47 | -2.43 | -2.62 | E4 |  | 4.94 | 1.79 | 0.86 | 4.85 | -2.53 | -3.18 | E4 |
| qPH56-H-1 | H | Satt181-Sat_334 |  |  |  |  |  |  |  |  | 3.13 | 8.23 | 4.57 | 2.09 | -1.83 | -4.82 | E3 |
| qPH56-H-2 | H | Satt253-Satt469 | 3.26 | 9.03 | 1.09 | 2.83 | -5.99 | 2.07 | E4 |  |  |  |  |  |  |  |  |
| qPH56-H-3 | H | Satt293-Sat_180 |  |  |  |  |  |  |  |  | 3.91 | 5.84 | 1.93 | 2.09 | -2.05 | -1.97 | E2 |
| qPH56-J-1 | J | Sat_224-Sat_396 |  |  |  |  |  |  |  |  | 4.45 | 6.63 | 2.52 | -1.44 | -2.72 | 1.65 | E2 |
| qPH56-J-2 | J | Sat_228-Satt431 | 3.05 | 6.04 | -3.05 | 1.31 | -2.22 | 3.96 | E4 |  |  |  |  |  |  |  |  |
| qPH56-K-1 | K | Satt727-Satt055 |  |  |  |  |  |  |  |  | 6.08 | 9.80 | -1.98 | 3.08 | -3.83 | 2.74 | E2 |
| qPH56-L-1 | L | Satt156-Satt664 |  |  |  |  |  |  |  |  | 3.57 | 8.13 | 2.90 | 0.53 | -2.06 | -1.38 | E1 |
| qPH56-M-1 | M | Satt245-Satt677 | 3.44 | 12.04 | 5.78 | -13.96 | 5.01 | 3.16 | E2 |  |  |  |  |  |  |  |  |
|  |  |  | 3.18 | 14.83 | -0.71 | -10.67 | 3.97 | 7.40 | E3 |  |  |  |  |  |  |  |  |
| qPH56-N-1 | N | Satt022-Satt257 |  |  |  |  |  |  |  |  | 3.30 | 6.65 | 3.15 | 0.57 | -1.57 | -2.15 | E2 |
| qPH56-N-2 | N | Satt125-Satt624 | 6.68 | 12.06 | 6.27 | 3.26 | -8.78 | -0.74 | E2 |  |  |  |  |  |  |  |  |
| qPH56-N-3 | N | Satt641-Sat_266 | 3.34 | 7.48 | -3.75 | -0.09 | 9.56 | -5.72 | E1 |  |  |  |  |  |  |  |  |
| qPH56-O-1 | O | BARCSOYSSR_10_0066-Satt487 |  |  |  |  |  |  |  |  | 3.52 | 6.20 | 2.74 | -1.07 | 0.04 | -1.71 | E1 |
|  |  |  | 3.79 | 7.57 | 4.86 | -3.30 | -2.87 | 1.32 | E4 |  |  |  |  |  |  |  |  |
| qPH56-O-2 | O | Sat_132-Satt358 |  |  |  |  |  |  |  |  | 3.70 | 7.49 | -3.71 | 0.55 | 3.20 | -0.04 | E2 |
| qPH63-A2-1 | A2 | Sat_115-Satt341 |  |  |  |  |  |  |  |  | 3.27 | 5.80 | -2.58 | -1.08 | 3.52 | 0.13 | E1 |
| qPH63-B2-1 | B2 | Sat_342-Sat_264 | 4.22 | 4.13 | -6.91 | -0.02 | 4.11 | 2.82 | E4 |  | 4.26 | 5.39 | -2.76 | -2.38 | 4.42 | 0.72 | E4 |
| qPH63-C2-1 | C2 | GMAC7L-Satt291 |  |  |  |  |  |  |  |  | 3.55 | 7.27 | -2.46 | -1.41 | -0.86 | 4.73 | E1 |
| qPH63-C2-2 | C2 | Satt643-Satt363 | 3.40 | 5.24 | 4.02 | 0.43 | -9.09 | 4.64 | E2 |  |  |  |  |  |  |  |  |
| qPH63-D1b-1 | D1b | Sat_135-Satt537 |  |  |  |  |  |  |  |  | 3.29 | 4.95 | -2.23 | -0.84 | 4.19 | -1.12 | E1 |
| qPH63-D1b-2 | D1b | Satt579-Satt290 | 3.21 | 7.09 | 2.27 | -10.07 | 4.99 | 2.81 | E2 |  |  |  |  |  |  |  |  |
| qPH63-D2-1 | D2 | Sat_222-Satt582 | 4.12 | 16.57 | -4.78 | -2.68 | 8.61 | -1.16 | E3 |  |  |  |  |  |  |  |  |
| qPH63-D2-2 | D2 | Sct_192-Sat_333 |  |  |  |  |  |  |  |  | 3.06 | 4.77 | 1.00 | -1.16 | 2.68 | -2.52 | E2 |
| qPH63-G-1 | G | Sat_141-Satt309 |  |  |  |  |  |  |  |  | 3.12 | 2.82 | 3.03 | 0.70 | -1.47 | -2.26 | E4 |
| qPH63-G-2 | G | Satt288-Sct_199 | 3.32 | 3.27 | 5.45 | 3.74 | -3.88 | -5.32 | E1 |  | 3.19 | 4.60 | 2.53 | 1.40 | -1.11 | -2.81 | E1 |
| qPH63-H-1 | H | BARCSOYSSR_12_0187-Satt293 |  |  |  |  |  |  |  |  | 4.21 | 5.64 | 4.19 | -1.85 | 0.21 | -2.54 | E3 |
| qPH63-H-2 | H | Sat_158-Satt181 |  |  |  |  |  |  |  |  | 3.30 | 11.99 | -4.90 | 9.87 | -1.99 | -2.98 | E4 |
| qPH63-H-3 | H | Satt253-Satt469 |  |  |  |  |  |  |  |  | 6.01 | 12.15 | 2.59 | 3.59 | -3.29 | -2.88 | E2 |
| qPH63-M-1 | M | Satt245-Satt677 | 3.71 | 11.08 | 7.32 | -22.95 | 8.25 | 7.38 | E2 |  |  |  |  |  |  |  |  |
| qPH63-M-2 | M | Satt336-Satt346 |  |  |  |  |  |  |  |  | 3.53 | 5.67 | 1.42 | -3.40 | 3.44 | -1.46 | E3 |
| qPH63-M-3 | M | Satt346-Sat_121 |  |  |  |  |  |  |  |  | 3.60 | 7.42 | -0.12 | -2.45 | 4.76 | -2.19 | E3 |
| qPH63-N-1 | N | Satt125-Satt624 | 3.88 | 5.54 | 6.09 | 2.57 | -9.71 | 1.04 | E2 |  |  |  |  |  |  |  |  |
| qPH63-N-2 | N | Satt159-Sct_195 |  |  |  |  |  |  |  |  | 5.18 | 12.56 | -0.65 | 7.06 | -3.19 | -3.21 | E3 |
| qPH63-O-1 | O | Sat_196-BARCSOYSSR_10_0066 | 3.18 | 4.66 | 6.16 | -0.86 | -9.66 | 4.36 | E1 |  |  |  |  |  |  |  |  |
|  |  |  | 3.20 | 3.97 | 4.81 | 0.69 | -8.27 | 2.77 | E4 |  |  |  |  |  |  |  |  |
| qPH70-A2-1 | A2 | Sat_115-Satt341 |  |  |  |  |  |  |  |  | 3.65 | 1.87 | 2.13 | -2.96 | -2.77 | 3.61 | E1 |
| qPH70-C1-1 | C1 | Sat_207-Satt338 |  |  |  |  |  |  |  |  | 3.43 | 1.10 | -2.25 | 4.25 | -2.14 | 0.14 | E3 |
| qPH70-C1-2 | C1 | Satt195-Sat_042 | 4.08 | 6.47 | -4.83 | 6.73 | -5.10 | 3.20 | E2 |  |  |  |  |  |  |  |  |
| qPH70-C2-1 | C2 | Satt643-Satt363 | 3.17 | 6.50 | 7.26 | -0.24 | -11.25 | 4.23 | E1 |  |  |  |  |  |  |  |  |
|  |  |  | 3.40 | 4.50 | 4.76 | 1.08 | -8.39 | 2.55 | E2 |  |  |  |  |  |  |  |  |
| qPH70-D1b-1 | D1b | Sat_373-Satt701 | 3.08 | 5.18 | 10.45 | -3.28 | -1.08 | -6.09 | E1 |  |  |  |  |  |  |  |  |
| qPH70-D1b-2 | D1b | Satt546-Satt703 |  |  |  |  |  |  |  |  | 3.45 | 1.92 | -0.96 | -1.91 | 5.15 | -2.28 | E1 |
| qPH70-D1b-3 | D1b | Satt579-Satt290 | 3.08 | 7.94 | 0.31 | -9.22 | 7.97 | 0.93 | E2 |  |  |  |  |  |  |  |  |
| qPH70-D2-1 | D2 | Sat_222-Satt582 | 4.03 | 6.12 | -5.13 | -3.06 | 9.11 | -0.93 | E3 |  |  |  |  |  |  |  |  |
| qPH70-E-1 | E | Satt699-Sat_381 |  |  |  |  |  |  |  |  | 7.60 | 4.79 | 11.71 | -4.81 | -3.91 | -2.99 | E3 |
| qPH70-F-1 | F | AW186493-Satt659 | 3.35 | 6.02 | -1.18 | 4.21 | 5.08 | -8.11 | E2 |  |  |  |  |  |  |  |  |
| qPH70-F-2 | F | Satt595-AW756935 |  |  |  |  |  |  |  |  | 7.98 | 4.71 | -4.33 | 12.49 | -4.72 | -3.44 | E3 |
| qPH70-G-1 | G | Satt288-Sct_199 | 5.20 | 6.70 | 7.10 | 6.56 | -5.74 | -7.92 | E1 |  | 3.43 | 2.73 | 0.09 | 6.00 | -2.38 | -3.71 | E1 |
|  |  |  | 5.20 | 7.03 | 5.75 | 5.65 | -4.34 | -7.06 | E2 |  | 7.33 | 8.51 | 1.32 | 3.46 | -2.49 | -2.29 | E2 |
|  |  |  | 4.12 | 7.01 | 3.33 | 7.72 | -3.81 | -7.25 | E3 |  | 9.64 | 5.56 | 11.52 | -3.72 | -3.80 | -4.00 | E3 |
| qPH70-H-1 | H | Sat_158-Satt181 |  |  |  |  |  |  |  |  | 3.95 | 7.94 | -1.08 | 4.89 | -1.80 | -2.00 | E2 |
| qPH70-K-1 | K | Satt055-BARCSOYSSR_09_0183 |  |  |  |  |  |  |  |  | 10.41 | 4.67 | -3.82 | -4.50 | 12.59 | -4.27 | E3 |
| qPH70-M-1 | M | Satt245-Satt677 | 3.10 | 12.11 | -4.78 | -9.82 | 4.63 | 9.97 | E3 |  |  |  |  |  |  |  |  |
| qPH70-N-1 | N | Satt641-Sat_266 |  |  |  |  |  |  |  |  | 3.97 | 4.64 | -3.72 | -3.87 | 11.15 | -3.56 | E3 |
| qPH70-O-1 | O | BARCSOYSSR_10_1172-BARCSOYSSR_10_0581 |  |  |  |  |  |  |  |  | 8.47 | 5.13 | -4.57 | -5.71 | 12.33 | -2.05 | E3 |
| qPH70-O-2 | O | Satt487-Satt592 |  |  |  |  |  |  |  |  | 9.17 | 4.76 | -3.65 | 10.62 | -3.80 | -3.17 | E3 |

^1^ E1 represents sowing seed on May 3 in 2014; E2 represents sowing seed on May 25 in 2014; E3 represents sowing seed on May 9 in 2015; E4 represents sowing seed on May 25 in 2015.
